# Acute myeloid leukemia cells secrete microRNA-4532-containing exosomes to mediate normal hematopoiesis in hematopoietic stem cells by activating the LDOC1-dependent STAT3 signaling pathway

**DOI:** 10.1186/s13287-019-1475-7

**Published:** 2019-12-16

**Authors:** Chen Zhao, Feng Du, Yang Zhao, Shanshan Wang, Ling Qi

**Affiliations:** 1 Department of Clinical Hematology, Jilin Medical University, Jilin, 132013 People’s Republic of China; 2 Department of Pathogenic Biology, Jining Medical University, Jining, Jining, 272067 People’s Republic of China; 3 Department of Infectious Disease, No. 965 Hospital of PLA Joint Logistic Support Force, Jilin, 132013 People’s Republic of China; 4 Key Laboratory of Precision Oncology of Shandong Higher Education, Institute of precision medicine, Jining Medical University, Jining, 272067 People’s Republic of China; 5 Department of Pathophysiology, Jilin Medical University, No. 5, Jilin Street, Jilin, 132013 Jilin Province People’s Republic of China; 6 The Sixth Affiliated Hospital of Guangzhou Medical University, Qingyuan People’s Hospital, B24 Yinquan South Road, Qingyuan, 511518 Guangdong Province People’s Republic of China

**Keywords:** Acute myeloid leukemia, Exosomes, miR-4532, LDOC1, STAT3 signaling pathway, Hematopoiesis, Hematopoietic stem cells

## Abstract

**Background:**

MicroRNA (miR)-containing exosomes released by acute myeloid leukemia (AML) cells can be delivered into hematopoietic progenitor cells to suppress normal hematopoiesis. Herein, our study was performed to evaluate the effect of exosomal miR-4532 secreted by AML cells on hematopoiesis of hematopoietic stem cells.

**Methods:**

Firstly, differentially expressed miRs related to AML were identified using microarray analysis. Subsequently, AML cell lines were collected, and CD34^+^ HSCs were isolated from healthy pregnant women. Then, miR-4532 expression was measured in AML cells and AML cell-derived exosomes and CD34^+^ HSCs, together with evaluation of the targeting relationship between miR-4532 and LDOC1. Then, AML cells were treated with miR-4532 inhibitor, and exosomes were separated from AML cells and co-cultured with CD34^+^ HSCs. Gain- and loss-function approaches were employed in CD34^+^ HSCs. Colony-forming units (CFU) and expression of dickkopf-1 (DKK1), a hematopoietic inhibiting factor associated with pathogenesis of AML, were determined in CD34^+^ HSCs, as well as the extents of JAK2 and STAT3 phosphorylation and LDOC1 expression.

**Results:**

miR-4532 was found to be upregulated in AML cells and AML cell-derived exosomes, while being downregulated in CD34^+^ HSCs. In addition, exosomes released by AML cells targeted CD34^+^ HSCs to decrease the expression of CFU and increase the expression of DKK1. miR-4532 was delivered into CD34^+^ HSCs to target LDOC1 via AML cell-released exosomes. AML cell-derived exosomes containing miR-4532 inhibitor increased CFU but reduced DKK1 in CD34^+^ HSCs. Inhibition of miR-4532 or JAK2, or ectopic expression of LDOC1 upregulated CFU and downregulated DKK1 expression as well as the extents of JAK2 and STAT3 phosphorylation in CD34^+^ HSCs.

**Conclusion:**

In conclusion, AML cell-derived exosomes carrying miR-4532 repress normal HSC hematopoiesis via activation of the LDOC1-dependent STAT3 signaling pathway.

## Introduction

Acute myeloid leukemia (AML) is a cancer of the blood cells in which too many myeloid cells (a type of white blood cell) are made by the bone marrow [1]. In addition, as a type of genetical heterogeneous clonal disease, AML is featured by increases in somatically acquired genetic alterations in hematopoietic progenitor cells that affect normal self-renewal, proliferation, and differentiation, all of which are implicated in the process of hematopoiesis [2]. The self-renewal, proliferation, and differentiation of hematopoietic stem cells (HSCs) are involved in hematopoiesis [3]. Moreover, a prior study highlighted that allogeneic HSC transplantation serves an effective treatment method for patients with secondary AML [4]. It is commonly acknowledged that exosomes are secreted by a variety of cells such as tumor cells, and tumor-released exosomes can induce tumor growth and metastasis [5].

Exosomes can carry proteins, microRNAs (miRNAs), long noncoding RNA (lncRNA), and messenger RNAs (mRNAs) and participate in intracellular communication and several physiological and pathological cellular processes [6, 7]. Moreover, it has been documented that exosomes derived from blasts exert an inhibitory effect on hematopoiesis in AML [8]. Similarly, exosomes have also been highlighted as promising therapeutic targets of minimal residual disease in AML [9]. Meanwhile, tumor-derived exosomes repress functions of immune cells and associate with progression of solid tumor or AML [10]. In addition, it has been supported that AML cell-secreted exosomes deliver miRNAs into hematopoietic progenitor cell (HPC) to suppress hematopoiesis [8]. Furthermore, aberrant miRs have also been implicated in various hematologic malignancies like AML [11]. A study conducted by Feng et al. demonstrated the promoting role of miR-4532 overexpression in drug resistance of breast cancer cells [12]. Moreover, miR-4532 was predicted to possess putative binding site of the leucine zipper downregulated in cancer-1 (LDOC1) gene by online prediction software. Additionally, LDOC1 is downregulated in chronic lymphocytic leukemia (CLL) tissues and serves as a predictor of overall survival of untreated patients [13]. Another study demonstrated that as a novel inhibitor of signal transducer and activator of transcription 3 (STAT3), LDOC1 silencing increased the extents of Janus-activated kinase 2 (JAK2) and STAT3 phosphorylation [14]. Increased expression of STAT3 has also been documented in AML cells [15]. Based on the discussion above, we proposed a hypothesis that AML cell-derived exosomal miR-4532 might regulate HSC function via the LDOC1-dependent STAT3 signaling pathway. Therefore, the current study aims to examine the effect of AML cell-derived exosomes carrying miR-4532 on HSCs via the STAT3 signaling pathway by regulating LDOC1. Insights into the underlying mechanisms might offer a better understanding of hematopoiesis in AML, thus providing more effective therapeutic strategies for AML patients.

## Materials and methods

### Ethics statement

The current study and all protocols were approved by the Ethics Committee of Jilin Medical University. Signed informed consents were obtained from all patients prior to blood collection.

### Bioinformatics analysis

Firstly, the AML-related miR microarray data, GSE85769, was retrieved from the Gene Expression Omnibus database (GEO, https://www.ncbi.nlm.nih.gov/geo/), wherein CD34^+^ hematopoietic stem cells (HSCs) and exosomes were compared with AML cells and exosomes. Following the standardization pretreatment of data using the affy package of R language [16], the differentially expressed miRs were retrieved with the limma package [17]. The adjusted *p* value was expressed as adj.*p*.Val, and adj.*p*.Val < 0.05 was set as the standard for difference analysis. Subsequently, the heat map of differentially expressed miRs was plotted. Next, the downregulated genes were screened from AML-related microarray data GSE9476. Then, the target genes of differentially expressed miRs were predicted using TargetScan (http://www.targetscan.org/vert_71/), miRWalk (http://mirwalk.umm.uni-heidelberg.de/), RAID (http://www.rna-society.org/raid/index.html), and miRSearch (http://www.exiqon.com/microrna-target-prediction) combined with the downregulated genes. Finally, the intersection of the obtained genes was obtained using the Venn website (http://bioinformatics.psb.ugent.be/webtools/Venn/).

### Cell culture

Firstly, AML cell lines HL-60 (homozygous for TP53 deletion) [18], Molm-14 (carrying tandem duplication of fms-like tyrosine kinase-3 (FLT3) and mutation of calcineurin B-like proteins (CBL)) [19], and OCI-AML3 (harboring nucleophosmin (NPM1) mutation type A, NPM1-mA) [20] were purchased from Bena Culture Collection (Beijing, China). In addition, the ML-2 (KMT2A-MLLT4) [21] cell line was obtained from the German Collection of Microorganisms and Cell Cultures (Braunschweig, Germany). All aforementioned cell lines were cultured in Roswell Park Memorial Institute (RPMI) medium (Gibco, Grand Island, NY, USA) containing 10% fetal bovine serum (FBS, Atlanta Biologicals, Flowery Branch, GA, USA) and 1 × penicillin/streptomycin (Gibco, Grand Island, NY, USA) [22]. After culture, the cells were incubated at 37 °C with 5% CO_2_ and > 95% humidity.

Cord blood samples were obtained from healthy pregnant women in the Gynecology and Obstetrics Department of the Department of Pathophysiology, Jilin Medical University. All pregnant women underwent full-term normal deliveries without acute or chronic infections, hematological disorders, and hereditary history. Subsequently, cord blood mononuclear cells (CBMCs) were isolated using the Ficoll® density gradient medium (GE Healthcare, Roosendaal, The Netherlands), and CD34^+^ HSCs were sorted using magnetic beads according to manufacturer’s instructions (Miltenyi Biotec, Utrecht, The Netherlands). Afterwards, CD34^+^ HSCs were incubated in serum-depleted stem cell culture medium II (Sigma-Aldrich Chemical Company, St Louis, MO, USA) supplemented with l-glutamine, penicillin/streptomycin/amphotericin B (100 U/mL, Lonza, Verviers, Belgium), interleukin-3 (IL-3, 10 ng/mL), and stem cell factor (SCF, 50 ng/mL). Finally, the number of cells was counted using the trypan blue dye and the viability was assessed and recorded. All cells were cultured at 37 °C with 5% CO_2_ and 95% humidity [23].

### Isolation, identification, and labeling of exosomes from AML cells

**Fig. 1:**
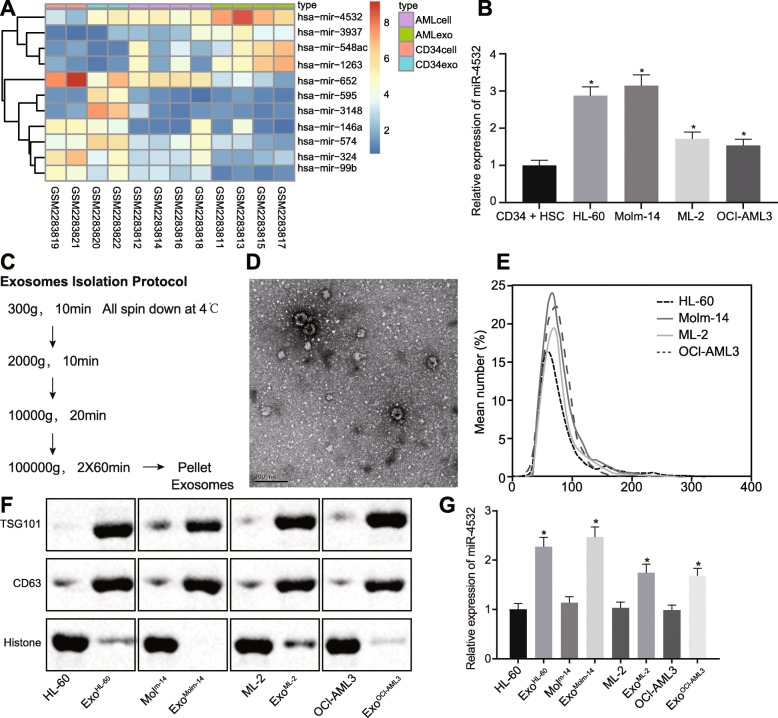
miR-4532 expression is upregulated in AML cell-secreted exosomes. **a** Screening of AML-related miRs in the GSE85769 microarray data. The *X* axis represents the sample number, and the *Y* axis represents the gene name. The histogram on the upper right is color gradation, with each rectangle representing a corresponding sample expression value. **b** miR-4532 expression in AML cell lines (HL-60, Molm-14, ML-2, and OCI-AML3) and CD34^+^ HSCs, as measured by RT-qPCR. *p < 0.05 vs. CD34^+^ HSCs. **c** Schematic diagram of exosome separation procedure. To exclude the effect of exosomes on bovine, the medium or FBS was centrifuged at 100,000×*g* for 10 h to remove the bovine exosomes. After that, the centrifugal medium was filtered through a 0.2-μm filter and collected for cell culture. AML cell line was cultured with the centrifugal medium. After 48 h, the supernatant was obtained. The process of exosome separation is shown in **c**. Finally, the purified exosomes were rinsed twice with PBS. **d** Morphology of exosomes observed under TEM. **e** The size and concentration of exosomes evaluated by nanoparticle tracking analysis. **f** Identification of marker exosomes (TSG101, CD63, and histone) by Western blot analysis. **g** miR-4532 expression in AML cell lines (HL-60, Molm-14, ML-2, and OCI-AML3) and exosomes secreted from AML cell lines (HL-60, Molm-14, ML-2, and OCI-AML3), as measured by RT-qPCR. *p < 0.05 vs. HL-60 cells, Molm-14 cells, ML-2 cells, or OCI-AML3 cells. Measurement data were described as mean ± standard deviation. Comparisons between two groups were analyzed by unpaired *t* test, and comparisons among multiple groups were analyzed by one-way analysis of variance (ANOVA), followed by Tukey’s post hoc test. The cell experiment was repeated three times to obtain the mean value. miR-4532, microRNA-4532; RT-qPCR, reverse transcription quantitative polymerase chain reaction; TEM, transmission electron microscope; PBS, phosphate buffer saline; FBS, fetal bovine serum

To exclude the effect of exosomes on bovine, the medium or FBS was centrifuged at 100,000×*g* for 10 h to remove the bovine exosomes. After that, the centrifugal medium was filtered using a 0.2-μm filter and collected for cell culture. Next, the AML cell line was cultured with the centrifugal medium, and the supernatant was obtained after 48 h. The process of exosome separation is shown in Fig. 1c. Finally, the purified exosomes were rinsed twice with phosphate buffer saline (PBS) [22].

A transmission electron microscope (TEM) was employed to observe and identify the morphology of exosomes, and the concentration and size of exosomes were evaluated by nanoparticle tracking analysis. The separated exosomes were diluted at the ratio of 1:10 and then observed using a Nanosight NS300 nanoparticle detector (Malvern, Westborough, MA, USA). Next, the exosomes were dissolved in radioimmunoprecipitation assay (RIPA) buffer, and the contents of proteins in exosomes were quantified using bicinchoninic acid (BCA) protein analysis kits (Thermo Fisher Scientific, Rockford, IL, USA). Western blot analysis was performed using the following antibodies: tumor susceptibility gene 101 (TSG101) antibody (ab125011, dilution ratio of 1:1000), CD63 antibody (ab134045, dilution ratio of 1:1000), and Histone antibody (ab1791, dilution ratio of 1:1000) [22].

The exosomes were labeled with carboxyfluorescein diacetate succinimidyl ester (CFSE) at 37 °C for 30 min. The labeled exosomes were rinsed with PBS, and the excess dye was discarded by centrifugation at 100,000×*g* for 1 h [22]. For in vitro tracking analysis of exosomes, the tagged exosomes and CD34^+^ HSCs were co-cultured in stem cell culture medium II for 4 h. Afterwards, the exosomes were observed under an Olympus BX41 microscope equipped with a charge-coupled device (CCD) camera (Magnafire; Olympus, Melville, NY, USA).

### Cell treatment

Molm-14 cells and CD34^+^ HSCs were seeded in a 12-well plate 24 h prior to transfection. When cell confluence reached 70%, the Molm-14 cells were treated with inhibitor-NC (75 nM) or miR-4532 inhibitor (75 nM), and CD34^+^ HSCs were transfected with mimic-NC (100 nM), miR-4532 mimic (100 nM), miR-4532 mimic + overexpression (oe)-LDOC1 (100 nM), si-NC (70 nM), or si-LDOC1 (70 nM), or treated with dimethyl sulfoxide (DMSO, 0.5 Um, Sigma-Aldrich Chemical Company, St Louis, MO, USA) or AZD1480 (0.5 uM, Selleckchem Chemicals, Houston, TX, USA) [14]. After being treated, the cells were cultured at 37 °C with 5% CO_2_ for 6–8 h, and then, the medium was renewed. Next, the cells were cultured for 24–48 h. The exosomes were isolated from the treated Molm-14 cells. The inhibitor-NC, miR-4532 inhibitor, mimic-NC, miR-4532 mimic, si-NC, si-LDOC1, and oe-LDOC1 were purchased from Guangzhou RiboBio Co., Ltd. (Guangzhou, China).

### Colony-forming units (CFU) in culture assay

The exosomes isolated from Molm-14 cells were cultured with 1 × 10^6^ CD34^+^ HSCs. Next, CFU analysis was conducted with Human Methylcellulose Complete Media (R&D Systems, Minneapolis, MN, USA). After 48 h of co-culture, the fresh culture medium was renewed, and the CD34^+^ HSCs were plated into a 35-mm dish at a density of 5000 cells per dish. Then, the CD34^+^ HSCs were subjected to incubation at 37 °C with 5% CO_2_ and ≥ 95% humidity for 7 days. On the seventh day, the colonies were observed and counted under an optical microscope. The experiment was repeated three times to obtain the mean value [24].

### Reverse transcription quantitative polymerase chain reaction

Total RNA from the cells and exosomes was extracted using miRNeasy or RNeasy kits (Qiagen, Germantown, MD, USA). Reverse transcription of mRNA was conducted according to the instructions of SuperScript III First-Strand Synthesis kit (Invitrogen, Carlsbad, California, USA ) with oligo (dT) priming. The primers (Table 1) were synthesized by Guangzhou RiboBio Co., Ltd. (Guangzhou, China). Then, qPCR was performed with the ABI PRISM 7500 FAST Real-Time PCR System (BioTek, Winooski, Vermont, USA) using SYBR Green PCR kits (Applied Biosystems, Foster City, CA, USA). With glyceraldehyde-3-phosphate dehydrogenase (GAPDH) serving as the internal reference, the relative expression of the target gene was analyzed with the 2^-ΔΔCt^ method [25]. For miR quantification, TaqMan assay kits (Applied Biosystems, Foster City, CA, USA) were applied for reverse transcription and RT-qPCR, with U6 serving as the internal reference [24].

**Table 1:** The primer sequences for RT-qPCR

Genes	Forward	Reverse
miR-4532	5′-CCCACCCCTTGCCTATAATC-3′	5′-TTCAGGGTTGCTCTGTTCAA-3′
U6	5′-CTCGCTTCGGCAGCACA-3′	5′-AACGCTTCACGAATTTGCGT-3′
LDOC1	5′-ATGACGACGAAGACGACGA-3′	5′-GAGGGTCGAGGGCCTAATAA-3′
DKK1	5′-GGAGGAGGGCAACTGAAGGAC-3’	5′-CCTCTCCTTTATGCCAATACTCGC-3′
GAPDH	5′-GGTATCGTGGAAGGACTCATGAC-3’	5′-ATGCCAGTGAGCTTCCCGTTCAG-3′

*miR-4532* microRNA-4532, *LDOC1* leucine-zipper downregulated in cancer 1, *DKK1* dickkopf-1, *GAPDH* glyceraldehyde-3-phosphate dehydrogenase, *RT-qPCR* reverse transcription quantitative polymerase chain reaction

### Dual-luciferase reporter gene assay

Firstly, the sequence fragment of LDOC1 3′-untranslated region (3′UTR) was artificially synthesized and introduced into the psiCHECK-2 vector (Promega, Madison, WI, USA). The mutation sites in the complementary sequence of seed sequences were designed on the wild type (WT) of LDOC1. Subsequently, all luciferase reporter plasmids, such as LDOC1 3′UTR-WT and LDOC1 3′UTR-mutant (MUT), were obtained. All aforementioned plasmids were co-transfected with miR-4532 mimic (2 nM, Dharmacon, Lafayette, CO, USA) or mimic-NC (2 nM, Dharmacon, Lafayette, CO, USA) into 4 × 10^5^ HEK-293 T cells (CRL-1415, Shanghai Xin Yu Biotech Co., Ltd., Shanghai, China) and CD34^+^ HSCs, respectively. After 48 h, the cells were lysed and the luciferase activity was determined using Dual-Luciferase Reporter Assay kits (RG005, Beyotime Biotechnology, Shanghai, China) in the Glomax20/20 luminometer fluorescence detector (Promega, Madison, WI, USA). The experiment was repeated three times to obtain the mean value [24].

### Western blot assay

Total protein of cells was extracted using high-efficiency RIPA lysis buffer (R0010, Beijing Solarbio Science & Technology Co., Ltd., Beijing, China) in strict accordance with the instructions. The protein concentration was determined using a BCA kit (20201 ES76, Yeasen Company, Shanghai, China). Then, the protein was separated by polyacrylamide gel electrophoresis and then transferred to PVDF membranes by wet transfer method. Next, the membranes were blocked using 5% BSA at room temperature for 1 h and probed overnight at 4 °C with the following diluted primary antibodies: mouse anti-human GAPDH (ab9425, 1:2500, Abcam, Cambridge, UK), JAK2 (ab39636, 1:1000, Abcam, Cambridge, UK), p-JAK2 (ab195055, 1:1000, Abcam, Cambridge, UK), STAT3 (ab31370, 1:500, Abcam, Cambridge, UK), and p-STAT3 (ab86430, 1:500, Abcam, Cambridge, UK). The membranes were washed for three times (5 min/time) with Tris-buffered saline Tween-20 (TBST), and horseradish peroxidase (HRP)-labeled goat anti-rabbit immunoglobulin G (IgG) (ab205718, 1:20,000, Abcam, Cambridge, UK) was added for 1 h of incubation at room temperature. Then, ImageJ 1.48u software (National Institutes of Health) was performed for protein quantification analysis. The ratio of the gray value of the target band to GAPDH was representative of the relative protein expression. The experiment was repeated three times.

### Statistical analysis

Statistical analyses were performed using the SPSS 21.0 software (IBM Corp. Armonk, NY, USA). Measurement data were presented as mean ± standard deviation. If data conformed to normal distribution and homogeneity test of variance, unpaired *t* test was applied for comparisons between two groups in an unpaired design, and one-way analysis of variance (ANOVA) was performed to compare the data among multiple groups, followed by Tukey’s post hoc test. A value of *p* < 0.05 was considered to be statistically significant.

## Results

### miR-4532 is highly expressed in exosomes derived from AML cells

Analysis of the gene expression dataset GSE85769 using the R Language revealed that miR-4532 was downregulated in CD34^+^ HSCs and CD34^+^ HSC-derived exosomes, while being upregulated in AML cells and AML cell-secreted exosomes (Fig. 1a). Therefore, it was speculated that miR-4532 exhibited associations with the development of AML. To verify this, we determined the expression of miR-4532 in AML cell lines (HL-60, Molm-14, ML-2, and OCI-AML3) and CD34^+^ HSCs. It was found that miR-4532 expression was higher in AML cell lines in comparison with the CD34^+^ HSCs (*p* < 0.05; Fig. 1b). The results were consistent with the results from the microarray data. Then, exosomes secreted from AML cell lines were isolated, purified (Fig. 1c), and subjected to TEM, nanoparticle tracking analysis, and Western blot analysis. It was demonstrated that exosomes presented with saucer-shaped stereochemical structures with clear membrane under TEM (Fig. 1d). In addition, the results of nanoparticle tracking analysis determined that the average particle size of exosomes was approximately 100 nm (Fig. 1e). Moreover, Western blot analysis displayed the existence of exosomes markers (TSG101, CD63, and histone) in exosomes secreted by AML cell lines (Fig. 1f). Subsequently, analyses of the expression of miR-4532 in AML cell lines as well as the corresponding exosomes revealed that miR-4532 was upregulated in exosomes secreted from AML cell lines, as compared to AML cell lines (*p* < 0.05; Fig. 1g). Collectively, it was demonstrated that miR-4532 was overexpressed in AML cell-derived exosomes.

### AML cell-secreted exosomes mediate hematopoiesis by targeting HSCs

**Fig. 2:**
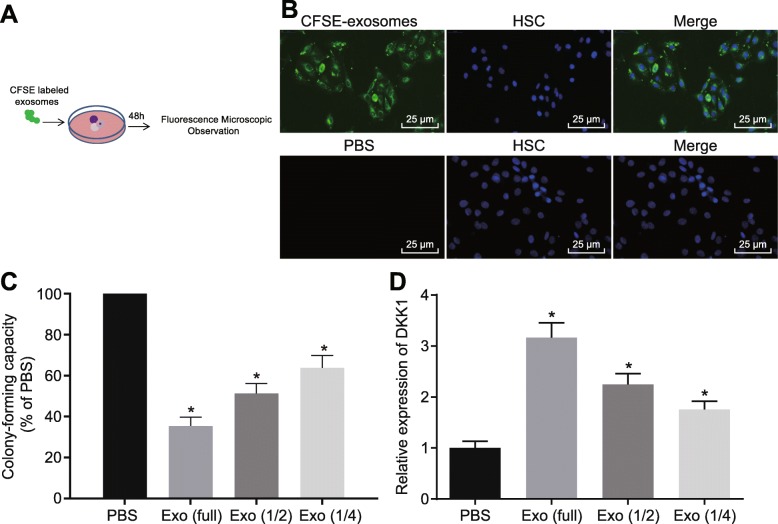
AML cell-secreted exosomes target HSCs to mediate hematopoiesis. **a**, **b** Uptake of CFSE-labeled exosomes by CD34^+^ HSCs, as detected under the fluorescence microscope. (×400) **c** Colony-forming capacity of CD34^+^ HSCs 48 h after culture with different dose of Molm-14 cell-derived exosomes. **d** Relative expression of hematopoietic suppressor gene DKK1 in CD34^+^ HSCs after culture with different dose of Molm-14 cell-derived exosomes measured by RT-qPCR. *p < 0.05 vs. the treatment of PBS. Measurement data were described as mean ± standard deviation. Comparisons between two groups were analyzed by unpaired *t* test. The cell experiment was repeated three times to obtain the mean value. miR-4532, microRNA-4532; LDOC1, leucine-zipper downregulated in cancer 1; DKK1, dickkopf-1; GAPDH, glyceraldehyde-3-phosphate dehydrogenase; RT-qPCR, reverse transcription quantitative polymerase chain reaction

Furthermore, we speculated that AML cell-derived exosomes were absorbed by HSCs to regulate hematopoiesis, thus correlating to the development of AML. To confirm the speculation, CFSE-labeled exosomes were co-cultured with CD34^+^ HSCs for 48 h, and exosomes were found to be colocalized with CD34^+^ HSCs (Fig. 2a, b). This finding suggested that AML cell-secreted exosomes might target HSCs. Next, to further explore the effect of exosomes from AML cells on HSCs, we performed colony-forming units (CFU) in culture assay. Compared with the PBS-treated CD34^+^ HSCs, the content of exosomes was found to be negatively correlated with CFU (Fig. 2c) and positively related with the relative expression of DKK1 (Fig. 2d). These results suggested that AML cell-secreted exosomes might regulate transcripts in HSCs to inhibit colony formation.

### AML cell-derived exosomes deliver miR-4532 to LDOC1

**Fig. 3:**
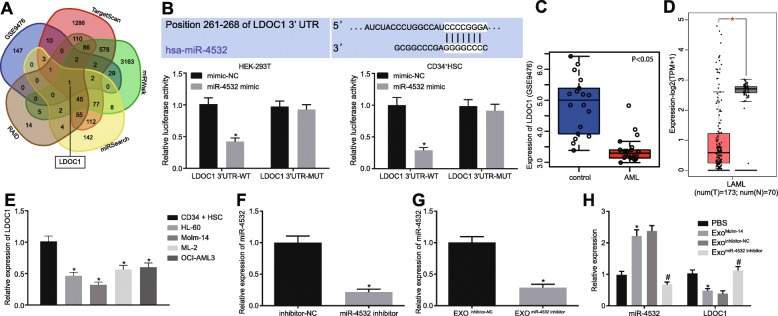
AML cells transfer miR-4532 by exosomes to target LDOC1. **a** Venn diagram of the predicted target genes of miR-4532. **b** The targeting relationship between miR-4532 and LDOC1 in HEK-293 T cells and CD34^+^ HSCs verified by dual-luciferase reporter gene assay (*p < 0.05 vs. the co-treatment of mimic-NC and LDOC1 3′-UTR WT). **c** LDOC1 expression in AML-related microarray data GSE9476. **d** LDOC1 expression in AML retrieved from the TCGA database (the *X* axis indicates the sample type, and the *Y* axis indicates the expression value; the red box indicates the tumor sample, and the gray box indicates the normal sample; *q < 0.01). **e** LDOC1 expression in AML cells and CD34^+^ HSCs measured by RT-qPCR (*p < 0.05 vs. CD34^+^ HSCs). **f** miR-4532 expression in Molm-14 cells after transfection with miR-4532 inhibitor measured by RT-qPCR (*p < 0.05 vs. Molm-14 cells cultured with miR-4532 inhibitor). **g** miR-4532 expression in the exosomes from Molm-14 cells measured by RT-qPCR (*p < 0.05 vs. exosomes from inhibitor-NC-treated Molm-14 cells). **h** miR-4532 and LDOC1 expression in CD34^+^ HSCs after co-culture with the exosomes from transfected Molm-14 cells measured by RT-qPCR (*p < 0.05 vs. CD34^+^ HSCs treated with PBS, ^#^*p* < 0.05 vs. CD34^+^ HSCs co-cultured with exosomes from inhibitor-NC-treated Molm-14 cells). Measurement data were described as mean ± standard deviation. Comparisons between two groups were analyzed by unpaired *t* test, and comparisons among multiple groups were analyzed by one-way analysis of variance (ANOVA), followed by Tukey’s post hoc test. The cell experiment was repeated three times to obtain the mean value. miR-4532, microRNA-4532; LDOC1, leucine-zipper downregulated in cancer 1; RT-qPCR, reverse transcription quantitative polymerase chain reaction

Firstly, the target genes of miR-4532 were predicted and the intersected genes were screened. It was found that LDOC1 was the only common target gene of predicted results, suggesting that miR-4532 might target LDOC1 (Fig. 3a). Dual-luciferase reporter gene assay results revealed that in HEK-293 T cells and CD34^+^ HSCs, the luciferase activity of LDOC1 3′-UTR WT was reduced in cells treated with miR-4532 mimic, while the luciferase activity of LDOC1 3′-UTR MUT did not differ significantly in cells treated with miR-4532 mimic relative to mimic-NC (Fig. 3b). In addition, the expression of LDOC1 in AML-related microarray data GSE9476 was determined, and it was found that LDOC1 was poorly expressed in AML cells (Fig. 3c). The expression of LDOC1 in AML was further retrieved in the TCGA database, which also showed downregulated LDOC1 expression (Fig. 3d). Next, LDOC1 expression was further determined in AML cells and CD34^+^ HSCs, which verified its poor expression in AML cells (Fig. 3e). In addition, RT-qPCR analysis demonstrated that LDOC1 expression in AML cells was negatively correlated with its expression in CD34^+^ HSCs (Fig. 3e). Because Molm-14 cell line presented with the highest expression of miR-4532, it was chosen as the study subject for subsequent experimentation. Molm-14 cells were cultured with miR-4532 inhibitor (Fig. 3f), and then, exosomes were isolated from Molm-14 cells. It was revealed that miR-4532 was downregulated in exosomes isolated from miR-4532 inhibitor-treated Molm-14 cells (Exo^miR-4532 inhibitor^) (Fig. 3g). The exosomes and CD34^+^ HSCs were co-cultured. The expression of miR-4532 was found to be increased, while that of LDOC1 was reduced in CD34^+^ HSCs co-cultured with the exosomes derived from the Molm-14 cells without any treatment (Exo^Molm-14^) (*p* < 0.05). However, compared with the treatment of inhibitor-NC-treated Molm-14 cells (Exo^inhibitor-NC^), miR-4532 was downregulated and LDOC1 was upregulated after undergoing treatment with Exo^miR-4532 inhibitor^ (*p* < 0.05; Fig. 3h). These results indicated that AML cells delivered miR-4532 to target LDOC1 via exosomes.

### AML cell-derived exosomal miR-4532 suppresses normal hematopoiesis by negatively regulating LDOC1

**Fig. 4:**
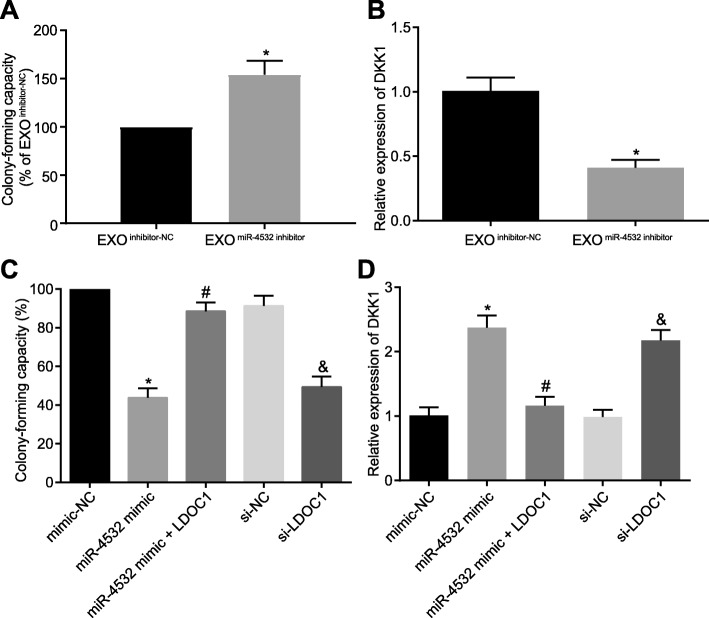
AML cell-derived exosomes overexpressing miR-4532 suppress normal hematopoiesis by targeting LDOC1. **a** The colony-forming capacity of CD34^+^ HSCs 48 h after culture with transfected Molm-14 cell-derived exosomes. **b** The relative expression of hematopoietic suppressor gene DKK1 in CD34^+^ HSCs after culture with transfected Molm-14 cell-derived exosomes measured by RT-qPCR. *p < 0.05 vs. CD34^+^ HSCs co-cultured with exosomes from inhibitor-NC-treated Molm-14 cells. **c** Colony-forming capacity of CD34^+^ HSCs after alteration of miR-4532 and LDOC1. **d** The relative expression of hematopoietic suppressor gene DKK1 in CD34^+^ HSCs after alteration of miR-4532 and LDOC1 measured by RT-qPCR. *p < 0.05 vs. CD34^+^ HSCs treated with mimic-NC, ^#^*p* < 0.05 vs. CD34^+^ HSCs cultured with miR-4532 mimic, ^&^*p* < 0.05 vs. CD34^+^ HSCs cultured with si-NC. Measurement data were described as mean ± standard deviation. Comparisons between two groups were analyzed by unpaired *t* test, and comparisons among multiple groups were analyzed by one-way analysis of variance (ANOVA), followed by Tukey’s post hoc test. The cell experiment was repeated three times to obtain the mean value. miR-4532, microRNA-4532; LDOC1, leucine-zipper downregulated in cancer 1; RT-qPCR, reverse transcription quantitative polymerase chain reaction

CD34^+^ HSCs were co-cultured with Exo^miR-4532 inhibitor^ to determine the relative expression of CFU and DKK1. As depicted in Fig. 4a, b, CD34^+^ HSCs co-cultured with Exo^miR-4532 inhibitor^ presented with increased CFU and downregulation of DKK1 relative to CD34^+^ HSCs co-cultured with Exo^inhibitor-NC^ (*p* < 0.05). Furthermore, we manipulated the expression of miR-4532 and LDOC1 in CD34^+^ HSCs to further clarify the mechanism. The treatment of miR-4532 mimic decreased CFU and upregulated the relative expression of DKK1. However, when LDOC1 was co-expressed, the CFU was found to be significantly increased, while the expression of DKK1 was reduced. In addition, LDOC1 silencing led to reduced CFU and notably elevated DKK1 in CD34^+^ HSCs (Fig. 4c, d). To sum up, it could be inferred that AML cell-derived exosomes inhibited normal hematopoiesis by negatively regulating LDOC1 via delivery of miR-4532.

### miR-4532 overexpression inhibits hematopoiesis of HSCs by activating the STAT3 signaling pathway via LDOC1

**Fig. 5:**
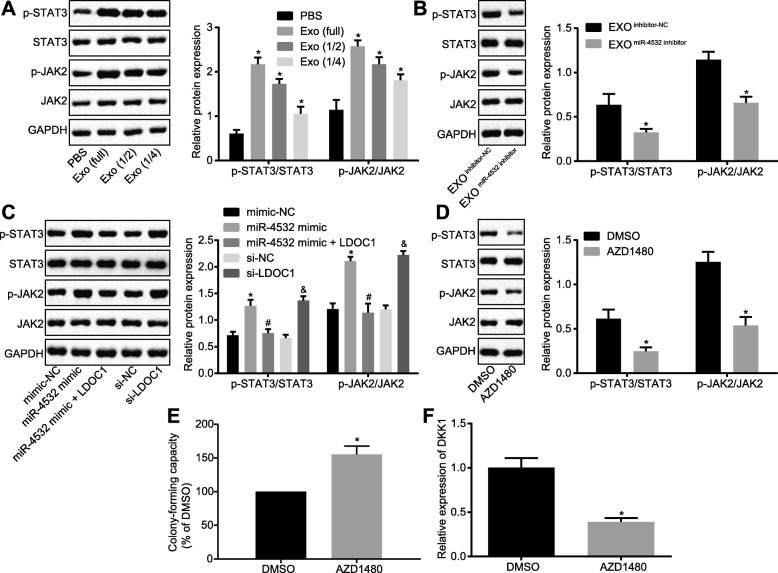
miR-4532 upregulation represses hematopoiesis of HSCs by activating LDOC1-dependent STAT3 signaling pathway. **a** The extents of JAK2 and STAT3 phosphorylation in CD34^+^ HSCs treated with different dose of exosomes, as determined by Western blot analysis (*p < 0.05 vs. CD34^+^ HSCs treated with PBS). **b** The extents of JAK2 and STAT3 phosphorylation in CD34^+^ HSC culture with transfected Molm-14 cell-derived exosomes, as determined by Western blot analysis (*p < 0.05 vs. CD34^+^ HSCs co-cultured with exosomes from inhibitor-NC-treated Molm-14 cells). **c** The extents of JAK2 and STAT3 phosphorylation in CD34^+^ HSCs after alteration of miR-4532 and LDOC1 determined by Western blot analysis (*p < 0.05 vs. CD34^+^ HSCs treated with mimic-NC, ^#^*p* < 0.05 vs. CD34^+^ HSCs cultured with miR-4532 mimic, ^&^*p* < 0.05 vs. CD34^+^ HSCs cultured with si-NC). **d** The extents of JAK2 and STAT3 phosphorylation in CD34^+^ HSCs after inhibition of JAK2 determined by Western blot analysis. **e** Colony-forming capacity of CD34^+^ HSCs after inhibition of JAK2. **f** The relative expression of hematopoietic suppressor gene DKK1 in CD34^+^ HSCs after inhibition of JAK2 measured by RT-qPCR. *p < 0.05 vs. CD34^+^ HSCs treated with DMSO. Measurement data were described as mean ± standard deviation. Comparison between two groups was analyzed by unpaired *t* test, and comparisons among multiple groups were analyzed by one-way analysis of variance (ANOVA), followed by Tukey’s post hoc test. The cell experiment was repeated three times to obtain the mean value. miR-4532, microRNA-4532; LDOC1, leucine-zipper downregulated in cancer 1; RT-qPCR, reverse transcription quantitative polymerase chain reaction; STAT3, signal transducer and activator of transcription 3

After elucidating that AML cell-derived exosomes inhibited normal hematopoiesis by negatively regulating LDOC1, we focused our attention to assess the relationship between miR-4532, LDOC1, and STAT3 signaling pathway. Western blot analysis was initially applied, which identified that the extents of JAK2 and STAT3 phosphorylation were positively correlated with the dose of exosomes (Fig. 5a). However, when CD34^+^ HSCs were co-cultured with Exo^miR-4532 inhibitor^, the extents of JAK2 and STAT3 phosphorylation were determined to be lower than that in CD34^+^ HSCs co-cultured with Exo^inhibitor-NC^ (*p* < 0.05; Fig. 5b). To investigate whether miR-4532 activated the STAT3 signaling pathway via LDOC1, we directly treated CD34^+^ HSCs with miR-4532 mimic or siRNA of LDOC1. Subsequently, the extents of JAK2 and STAT3 phosphorylation were found to be elevated in CD34^+^ HSCs with overexpressed miR-4532, while the contents were reduced as a result of co-expression of LDOC1. In addition, silencing of LDOC1 led to elevated extents of JAK2 and STAT3 phosphorylation (Fig. 5c). To study whether the STAT3 signaling pathway affected HSCs and hematopoiesis, CD34^+^ HSCs were treated with 0.5 μM AZD1480 (JAK2 inhibitor), and the extents of JAK2 and STAT3 phosphorylation were determined by Western blot analysis. The results showed that AZD1480 treatment reduced the extents of JAK2 and STAT3 phosphorylation (Fig. 5d). Additionally, CFU was found to be increased (Fig. 5e), while the relative expression of DKK1 was reduced (Fig. 5f) after CD34^+^ HSCs were treated with AZD1480. The aforementioned results displayed that miR-4532 upregulation inhibited hematopoiesis of HSCs by activating the STAT3 signaling pathway via downregulation of LDOC1 gene.

## Discussion

**Fig. 6:**
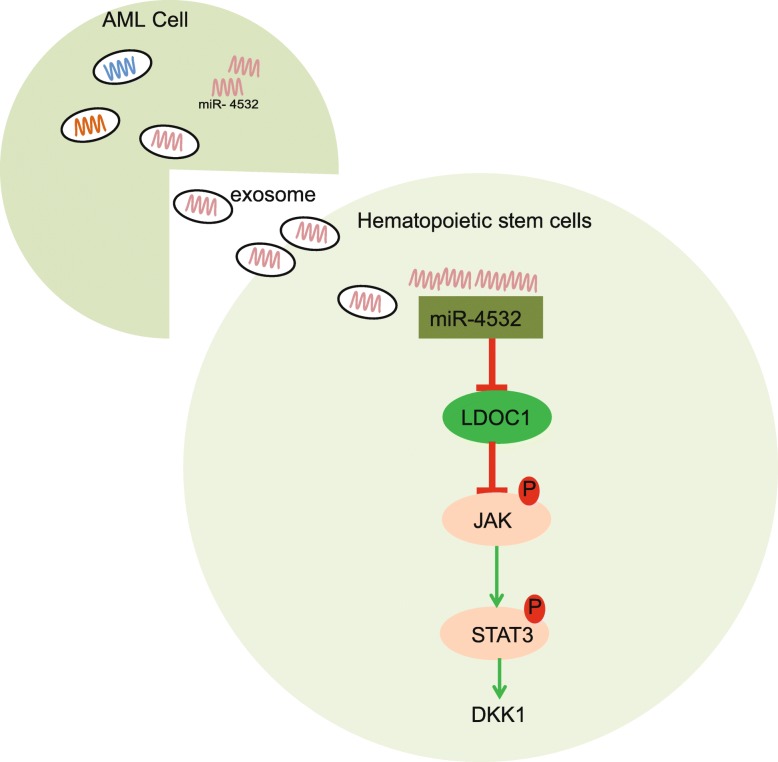
The mechanism of AML cell-secreted exosomal miR-4532 in hematopoiesis of HSCs. AML cell-secreted exosomes transfer miR-4532 into HSCs, and miR-4532 targets and downregulates LDOC1, thus activating the STAT3 signaling pathway. The activation of the STAT3 signaling pathway increased the hematopoietic suppressor gene DKK1 expression, thus suppressing hematopoiesis of HSCs. miR-4532, microRNA-4532; LDOC1, leucine-zipper downregulated in cancer 1; STAT3, signal transducer and activator of transcription 3

AML can arise in patients with underlying hematological conditions, or as a result of prior exposure to topoisomerases II, alkylating agents, or radiation [26]. Interestingly, given the expression of multiple myeloid markers in HSCs, it has been indicated HSCs could be employed in origin and targeted therapy of AML [27]. Moreover, another study demonstrated the emerging role of tumor-derived exosomes in the development of hematological malignancies and their high levels in plasma of AML patients [28]. Hence, the current study aimed to explore the effect of AML cell-derived exosomes on normal hematopoiesis of HSCs and the potential mechanism, hoping to seek novel targets for AML therapy. Consequently, we uncovered that exosomes derived from AML cells carrying miR-4532 inhibit the hematopoiesis of HSCs by activating the LDOC1-dependent STAT3 signaling pathway (Fig. 6).

Initially, we found that miR-4532 was upregulated, while LDOC1 was downregulated in AML cells and AML cell-secreted exosomes. Similarly, a prior study revealed that various miRs, such as miR-191 and miR-199, are overexpressed in AML samples [29]. Another study has also highlighted the restored expression of miR-29a in AML [30]. Furthermore, consistent with our results, miR-4532 is highly expressed in breast cancer [12]. Generally, miRs interact with their target genes at specific sites and regulate expression of respective target genes of miRs at a post-transcriptional level [31]. It was revealed in our study that LDOC1 was a target gene of miR-4532. Remarkably, it has been revealed that papillary thyroid carcinoma tissues exhibit decreased expression of LDOC1 versus the normal thyroid tissues [32]. In addition, the study conducted by Duzkale et al. identified downregulated expression of LDOC1 in the development of CLL [13]. Meanwhile, the current study also found that LDOC1 gene possesses the ability to negatively regulate the STAT3 signaling pathway. This is in accordance with the study conducted by Lee et al., wherein they highlighted that LDOC1 is an inhibitor of STAT3, and LDOC1 silencing causes an increase in the extents of JAK2 and STAT3 phosphorylation [14].

Exosomes are secreted by several cells, which can be transported between cells and transport proteins, miRs, lncRNA, and messenger RNAs (mRNAs) [6, 33]. Importantly, exosomes can also regulate the activation of target cells [34]. In the current study, we demonstrated that AML cell-released exosomes targeted HSCs and delivered miR-4532 into HSCs to activate the STAT3 signaling pathway by targeting LDOC1 gene, thus inhibiting normal hematopoiesis, as evidenced by increased DKK1 and decreased colony formation of CD34+ HSCs. Consistent with our results, another study reported that AML cell-secreted exosomes deliver miRs into HPCs to suppress hematopoiesis [8]. Meanwhile, it has also been documented that exosomal miRs derived from AML cells suppress the functioning of hematopoietic stem and progenitor cell (HSPC), inducing the loss of hematopoiesis by targeting c-MYB [24]. Furthermore, miR-150 also plays a suppressive role in hematopoietic recovery upon 5-fluorouracil-induced injury, which involves MYB [35]. Moreover, a recent study highlighted that the activity of HSCs could be disrupted by ectopic expression of a dominant negative form of STAT3 [36]. Subsequently, mechanistic investigation conducted by Siddiquee et al. revealed the constitutive activation of STAT3 in numerous human solid and hematological tumors [37]. At the same time, another study evidenced that as an oral inhibitor of STAT3, OPB-51602 can be applied in the treatment of relapsed and refractory hematological malignancies [38].

## Conclusion

To sum up, the current study evidenced that exosomes derived from AML cells carrying miR-4532 exert a suppressive effect on hematopoiesis of HSCs by the LDOC-dependent STAT3 signaling pathway, which facilitates a novel aspect of the treatment of AML patients. Our findings highlight miR-4532 and tumor-derived exosomal miRs as future alternative targets in therapy of AML. Furthermore, drug delivery by exosome may provide new ideas for AML therapy. However, the research is still at the preclinical stage and required more investigation into the role and mechanism of miR-4532 in AML. Besides, owing to the limitation of technology and expenditure, genetic perturbation such as CRSIPR knockout could not be utilized to further verify the inhibitor experiments. Thus, it is recommended that similar experiments are needed in order to further explore the intrinsic mechanisms.

## Data Availability

The datasets generated/analyzed during the current study are available.

## Abbreviations

3′UTR: 3′-Untranslated region
AML: Acute myeloid leukemia
ANOVA: Analysis of variance
CBL: Calcineurin B-like proteins
CCD: Charge-coupled device
CFU: Colony-forming units
CLL: Chronic lymphocytic leukemia
DKK1: Dickkopf-1
FLT3: fms-like tyrosine kinase-3
GAPDH: Glyceraldehyde-3-phosphate dehydrogenase
HPC: Hematopoietic progenitor cell
HSCs: Hematopoietic stem cells
JAK2: Janus-activated kinase 2
miRs: MicroRNAs
mRNAs: Messenger RNAs
NPM1: Nucleophosmin
TSG101: Tumor susceptibility gene 101
WT: Wild type

## References

[1] Klepin HD (2015). Pardee TSJHoCoNA. Acute Myeloid Leukemia.

[2] Marcucci G, Haferlach T, Dohner H (2011). Molecular genetics of adult acute myeloid leukemia: prognostic and therapeutic implications. J Clin Oncol.

[3] Mendelson A, Frenette PS (2014). Hematopoietic stem cell niche maintenance during homeostasis and regeneration. Nat Med.

[4] Lim Z (2010). Allogeneic hematopoietic stem-cell transplantation for patients 50 years or older with myelodysplastic syndromes or secondary acute myeloid leukemia. J Clin Oncol.

[5] Kahlert C, Kalluri R (2013). Exosomes in tumor microenvironment influence cancer progression and metastasis. J Mol Med (Berl).

[6] Barile L, Vassalli G (2017). Exosomes: therapy delivery tools and biomarkers of diseases. Pharmacol Ther.

[7] Kossinova OA (2017). Cytosolic YB-1 and NSUN2 are the only proteins recognizing specific motifs present in mRNAs enriched in exosomes. Biochim Biophys Acta, Proteins Proteomics.

[8] Boyiadzis M, Whiteside TL (2018). Exosomes in acute myeloid leukemia inhibit hematopoiesis. Curr Opin Hematol.

[9] Boyiadzis M, Whiteside TL (2016). Plasma-derived exosomes in acute myeloid leukemia for detection of minimal residual disease: are we ready?. Expert Rev Mol Diagn.

[10] Whiteside TL (2013). Immune modulation of T-cell and NK (natural killer) cell activities by TEXs (tumour-derived exosomes). Biochem Soc Trans.

[11] Marcucci G (2011). The prognostic and functional role of microRNAs in acute myeloid leukemia. Blood..

[12] Feng F (2018). Downregulation of hypermethylated in cancer-1 by miR-4532 promotes adriamycin resistance in breast cancer cells. Cancer Cell Int.

[13] Duzkale H (2011). LDOC1 mRNA is differentially expressed in chronic lymphocytic leukemia and predicts overall survival in untreated patients. Blood..

[14] Lee Chia-Huei, Yang Ji-Rui, Chen Chih-Yu, Tsai Ming-Hsien, Hung Pin-Feng, Chen Shin-Jih, Chiang Shang-Lun, Chang Han, Lin Pinpin (2019). Novel STAT3 Inhibitor LDOC1 Targets Phospho-JAK2 for Degradation by Interacting with LNX1 and Regulates the Aggressiveness of Lung Cancer. Cancers.

[15] Redell MS (2011). Stat3 signaling in acute myeloid leukemia: ligand-dependent and -independent activation and induction of apoptosis by a novel small-molecule Stat3 inhibitor. Blood..

[16] Gautier L (2004). Affy--analysis of Affymetrix GeneChip data at the probe level. Bioinformatics..

[17] Smyth GK (2004). Linear models and empirical bayes methods for assessing differential expression in microarray experiments. Stat Appl Genet Mol Biol.

[18] Wolf D, Rotter V (1985). Major deletions in the gene encoding the p53 tumor antigen cause lack of p53 expression in HL-60 cells. Proc Natl Acad Sci U S A.

[19] Quentmeier H (2003). FLT3 mutations in acute myeloid leukemia cell lines. Leukemia..

[20] Quentmeier H (2005). Cell line OCI/AML3 bears exon-12 NPM gene mutation-A and cytoplasmic expression of nucleophosmin. Leukemia..

[21] Drexler HG, Quentmeier H, MacLeod RA (2004). Malignant hematopoietic cell lines: in vitro models for the study of MLL gene alterations. Leukemia..

[22] Kumar B (2018). Acute myeloid leukemia transforms the bone marrow niche into a leukemia-permissive microenvironment through exosome secretion. Leukemia..

[23] Orsini Marion, Chateauvieux Sébastien, Rhim Jiyun, Gaigneaux Anthoula, Cheillan David, Christov Christo, Dicato Mario, Morceau Franck, Diederich Marc (2018). Sphingolipid-mediated inflammatory signaling leading to autophagy inhibition converts erythropoiesis to myelopoiesis in human hematopoietic stem/progenitor cells. Cell Death & Differentiation.

[24] Hornick NI (2016). AML suppresses hematopoiesis by releasing exosomes that contain microRNAs targeting c-MYB. Sci Signal.

[25] Liao FL (2018). Hematopoietic stem cell-derived exosomes promote hematopoietic differentiation of mouse embryonic stem cells in vitro via inhibiting the miR126/Notch1 pathway. Acta Pharmacol Sin.

[26] De Kouchkovsky I, Abdul-Hay M (2016). Acute myeloid leukemia: a comprehensive review and 2016 update. Blood Cancer J.

[27] Taussig DC (2005). Hematopoietic stem cells express multiple myeloid markers: implications for the origin and targeted therapy of acute myeloid leukemia. Blood..

[28] Boyiadzis M, Whiteside TL (2017). The emerging roles of tumor-derived exosomes in hematological malignancies. Leukemia..

[29] Garzon R (2008). MicroRNA signatures associated with cytogenetics and prognosis in acute myeloid leukemia. Blood..

[30] Han YC (2010). microRNA-29a induces aberrant self-renewal capacity in hematopoietic progenitors, biased myeloid development, and acute myeloid leukemia. J Exp Med.

[31] John B, Sander C, Marks DS (2006). Prediction of human microRNA targets. Methods Mol Biol.

[32] Zhao S (2015). LDOC1 inhibits proliferation and promotes apoptosis by repressing NF-kappaB activation in papillary thyroid carcinoma. J Exp Clin Cancer Res.

[33] Lasser C (2012). Exosomal RNA as biomarkers and the therapeutic potential of exosome vectors. Expert Opin Biol Ther.

[34] Mathivanan S, Ji H, Simpson RJ (2010). Exosomes: extracellular organelles important in intercellular communication. J Proteome.

[35] He Y, Jiang X, Chen J (2014). The role of miR-150 in normal and malignant hematopoiesis. Oncogene..

[36] Oh IH, Eaves CJ (2002). Overexpression of a dominant negative form of STAT3 selectively impairs hematopoietic stem cell activity. Oncogene..

[37] Al Zaid Siddiquee K, Turkson J (2008). STAT3 as a target for inducing apoptosis in solid and hematological tumors. Cell Res.

[38] Ogura M (2015). Phase I study of OPB-51602, an oral inhibitor of signal transducer and activator of transcription 3, in patients with relapsed/refractory hematological malignancies. Cancer Sci.

